# Number of nexin links detectable at standard electron microscopy of normal human nasal cilia and at nexin link deficiency

**DOI:** 10.1186/2046-2530-4-S1-P64

**Published:** 2015-07-13

**Authors:** U Stenram, C Cramnert

**Affiliations:** 1 University of Lund, Lund, Sweden

## Objective

We described 11 years ago 3 patients with missing nexin links as a possible cause of primary ciliary dyskinesia (PCD). The assumption was substantiated last year by finding a mutation in these patients. Here we counted the number of nexin links per cilium in normal cilia and at deficiency of nexin links.

## Methods

We counted the nexin links, inner (IDA) and outer (ODA) dynein arms and microtubuli in each of, if possible, 50 cilia in 41 patients with normal cilia, 4 patients with deficiency of nexin links only and 4 with deficiency of nexin links and IDA.

## Results

In the control group the median number of nexin links was 4.5 per cilium, range 3.4 - 5.3. In the second group the mean number of nexin links per cilium was 1.1 - 1.4, in the third group 0.8 - 1.2, per patient. The median number of IDA was in the control group 4.2, range 3.3 - 5.2. In groups 2 and 3 the numbers were 3.0 - 3.5 and 0.2 - 1.0, respectively, per patient. Numbers of ODA were normal in all groups.

## Conclusion

It is possible to reliably count the number of nexin links in nasal human cilia and to distinguish cases with missing nexin links from normal controls.

